# Comparison of Two Voltage-Sensitive Dyes and Their Suitability for Long-Term Imaging of Neuronal Activity

**DOI:** 10.1371/journal.pone.0075678

**Published:** 2013-10-04

**Authors:** Stephanie Preuss, Wolfgang Stein

**Affiliations:** 1 Institute of Neurobiology, Ulm University, Ulm, Germany; 2 School of Biological Sciences, Illinois State University, Normal, Illinois, United States of America; Claremont Colleges, United States of America

## Abstract

One of the key approaches for studying neural network function is the simultaneous measurement of the activity of many neurons. Voltage-sensitive dyes (VSDs) simultaneously report the membrane potential of multiple neurons, but often have pharmacological and phototoxic effects on neuronal cells. Yet, to study the homeostatic processes that regulate neural network function long-term recordings of neuronal activities are required. This study aims to test the suitability of the VSDs RH795 and Di-4-ANEPPS for optically recording pattern generating neurons in the stomatogastric nervous system of crustaceans with an emphasis on long-term recordings of the pyloric central pattern generator. We demonstrate that both dyes stain pyloric neurons and determined an optimal concentration and light intensity for optical imaging. Although both dyes provided sufficient signal-to-noise ratio for measuring membrane potentials, Di-4-ANEPPS displayed a higher signal quality indicating an advantage of this dye over RH795 when small neuronal signals need to be recorded. For Di-4-ANEPPS, higher dye concentrations resulted in faster and brighter staining. Signal quality, however, only depended on excitation light strength, but not on dye concentration. RH795 showed weak and slowly developing phototoxic effects on the pyloric motor pattern as well as slow bleaching of the staining and is thus the better choice for long-term experiments. Low concentrations and low excitation intensities can be used as, in contrast to Di-4-ANEPPS, the signal-to-noise ratio was independent of excitation light strength. In summary, RH795 and Di-4-ANEPPS are suitable for optical imaging in the stomatogastric nervous system of crustaceans. They allow simultaneous recording of the membrane potential of multiple neurons with high signal quality. While Di-4-ANEPPS is better suited for short-term experiments that require high signal quality, RH795 is a better candidate for long-term experiments since it has only minor effects on the motor pattern.

## Introduction

One of the challenges of modern Neuroscience is the simultaneous manipulation and activity measurement of many neurons to uncover how neural circuits and networks deliver their integral functionality [1]–[9]. Classical methods such as extracellular field potential recordings or single cell electrophysiology are, however, insufficient to fulfill the requirements for characterizing more than a few neurons. Optical imaging with Calcium- or voltage-sensitive dyes (VSDs) on the other hand, is well-suited for recording large sets of neurons with single neuron resolution, yet comes with different problems: Calcium dyes, for example, lack the appropriate time resolution to observe quick neural events and only act as indirect reporters for changes in membrane potential. In contrast, VSDs are typically much faster and directly report changes in membrane potential. They have been applied successfully for measuring population activities and, more rarely, to report the membrane potential of single cells [10]–[12] even though their signal-to-noise ratio (S/N) is smaller than that of intracellular recordings. With the exception of fluorescent proteins [13], dyes typically have to be bath-applied to bulk stain neurons [14]–[17], involving the risk of toxic influences of the dye or the solvent. During the experiment, VSDs often show phototoxic effects (during light exposure) and they bleach over time [17]–[20]. The specifics of the model organism used, such as for example the salinity of the extra- and intracellular medium, enzymatic activities and membrane properties might affect the binding characteristics or toxicity of the dye [21], [22]. Thus, dyes need to be evaluated separately for each system regarding their ability to stain neurons, their S/N, their bleaching kinetics and regarding their toxic and phototoxic effects.

Phototoxic effects in particular might be difficult to detect since many neuronal activities - although reproducible - are rather variable and controls are therefore difficult to achieve. For example, stimuli may be applied that elicit a certain neuronal response and this response is then recorded using VSDs. In that case, there is little to no control over the effects of the dye, since the uninfluenced response is unknown. Only if the response appears exceedingly different from previous stimuli (or the expected outcome), possible phototoxic effects are taken into account. Phototoxic effects increase with intensity and duration of the excitation light [19], [20], [23]. This Catch-22 situation can mostly be circumvented by using short imaging sessions of a few seconds. Yet, many neuronal processes last for longer (minutes to hours) and hence require continuous or repetitive monitoring of neuronal activity for longer time periods. The stomatogastric nervous system is especially suitable to test long-term effects, as analysis of spontaneous rhythmic activity patterns is very sensitive to changes and therefore even subtle effects can be detected (in contrast to when stimulus-evoked activity is used). In addition, the activity patterns produced in this system represent the motor output of a whole network of neurons, and thus reflect the healthiness of many neurons and their synaptic interactions.

Here, we report the comparison of two VSDs, their S/N, long-term (photo-) toxic effects and their ability to stain identified motor neurons in the crustacean stomatogastric nervous system. The neuronal circuits in the stomatogastric ganglion (STG) have been thoroughly characterized on a single cell level [24]–[26]. All STG neurons are identified and their intrinsic properties and connections are well known. They deliver their function quasi-autonomously, i.e. they form central pattern generators (CPGs; [25], [27], [28]) that drive the rhythmic movements of muscles in the foregut. The pyloric CPG is continuously active and controls the movements of the pyloric filter apparatus [25], [29]. Its motor output is highly stereotyped unless perturbed by sensory input or neuromodulators, and can be monitored using extracellular motor nerve recordings. The pyloric rhythm is triphasic and shows a stable phase relationship of the pyloric neurons over many hours. Its cycle period ranges from 0.5 to 2 seconds. The robustness of this rhythmic activity, the fact that it can be maintained in isolated ganglion preparations for hours, if not days, and the ability of the pyloric circuit to recover from injury have recently attracted several studies involving long-term neuronal recordings [30]–[32], emphasizing the need to monitor neuronal membrane potential over extended periods of time. Therefore, we are testing the dyes in this well characterized pattern generating circuit, which allows us to focus on long-term effects ranging from minutes to hours or days.

Our previous work has already demonstrated that VSD imaging allows simultaneous recording of many STG neurons over several seconds [16] and we could also show that there are no apparent toxic effects of the VSD during short-term application [33]. However, very little is known about phototoxicity and long-term effects of the dyes. Due to its stereotyped output the pyloric network offers the possibility to test different VSDs and their influence not only on single cells, but on a fully functional motor network. In contrast to other test-bed preparations, deviations from spontaneously occurring neuronal behavior can easily be detected.

Many promising VSDs have been described previously, in a variety of systems (e.g.: Di-2-ANEPPQ, *Helix* [11]; Di-4-ANEPPS, rabbit [34]; RH795, honey bee [35]; Di-8-ANEPPS, guinea pig [17]; FM4-64, *Aplysia* [36]; RH414, salamander [37], as well as several Chimeric dyes [17]). We used Di-4-ANEPPS, which provides a sufficiently good signal quality to record from multiple STG neurons [38] and RH795, which has been successfully used in other invertebrate ganglia [14] and tested them for their suitability for long-term recordings. We show that they possess toxic and phototoxic effects but vary prominently regarding their level of toxicity, their ability to stain neurons and their S/N. We conclude that Di-4-ANEPPS is well-suited for short-term recordings, whereas RH795 is a better candidate for long-term recordings.

## Materials and Methods

### Dissection

**Figure 1:**
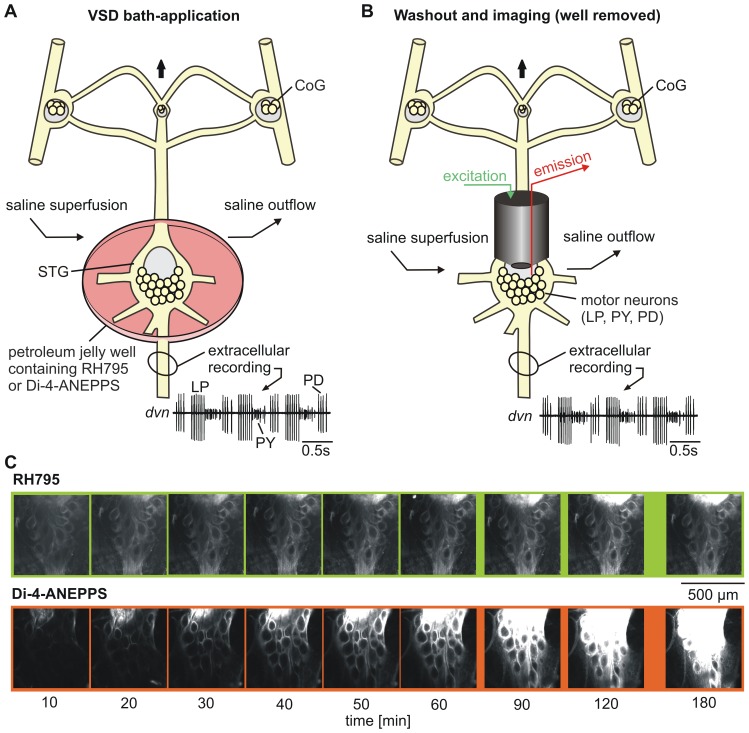
Experimental design and ganglion staining. A. Schematic of STNS showing experimental design for bath application of the dyes and electrophysiological recording of the triphasic pyloric rhythm (LP, PD and PY neurons). The STG was surrounded by a petroleum jelly well holding the VSD. An additional petroleum jelly well was placed around the *dvn* for extracellular recording. CoG, commissural ganglion; *dvn*, dorsal ventricular nerve; LP, lateral pyloric neuron; OG, oesophageal ganglion; PD, pyloric dilator neurons; PY, pyloric constrictor neurons; STG, stomatogastric ganglion. B. Similar to A, but after dye wash-out. The STG was continuously superfused with saline. VSDs were excited by green fluorescence light and red emission was recorded. C. Both dyes stained the STG in a time-dependent manner. Photos of the STG during bath-application of dyes at a concentration of 0.2 mM (top: RH795, bottom: Di-4-ANEPPS) at indicated times after dye application. The shutter speed of the camera was 200 msec for RH795 and 100 msec for Di-4-ANEPPS.

Adult *Cancer pagurus* L. were obtained from Feinfisch (Neu-Ulm, Germany) and *Cancer borealis* were delivered from “The Fresh Lobster” (Boston, MA, USA). Crabs were kept in tanks with artificial sea water (salt content ∼1.025 g/cm^3^) made from artificial sea salt (Reefsalt, AquaMed). Tanks were kept at a temperature of 9–12°C and a 12 hour light-dark cycle. Animals were anesthetized by packing them in ice for 20 min before dissection. We used isolated nervous systems to perform our experiments [39]. In summary, the STNS was pinned down in a silicone elastomer-lined (ELASTOSIL RT-601, Wacker, Munich, Germany) Petri dish and continuously superfused (7–12 ml/min) with chilled saline (10–13°C; Fig. 1B). Physiological saline consisted of (mMol*l-1): NaCl, 440; MgCl2, 26; CaCl2, 13; KCl, 11; trisma base, 10; maleic acid, 5 (pH 7.4–7.6).

### Extracellular Recording

During all experiments the neuronal activity on the main motor nerve, the dorsal ventricular nerve (*dvn*), was recorded (Figs. 1A,B). In some experiments the pyloric dilator nerve, which contains the pyloric dilator (PD) neurons, was recorded in addition. The PD neurons are part of the pacemaker ensemble of the pyloric circuit [24]. Extracellular recordings were performed by isolating a part of a nerve from the surrounding medium using a petroleum jelly well and subsequent measurements of field potential changes between two wires, one inside and one outside of the well. The differential signal was recorded, filtered and amplified with an AC differential amplifier (A-M Systems Modell 1700, Carlsborg, WA, USA). Files were recorded, saved and analyzed using Spike 2 Software (version 6.11 or 7.09; CED, Cambridge, UK). Signal analysis including measurements of instantaneous frequencies, signal amplitudes or the noise of the signal, as well as averaging of signals was performed using associated scripts for Spike2 (www.neurobiologie.de/spike2). For cycle-based averaging [16], the cycle period of the pyloric rhythm was defined as the duration between the onset of a PD neuron burst and the onset of the subsequent PD burst. Burst durations were defined as the time between the first and last action potential (spike) within a burst. Intraburst spike frequency was measured as the number of spikes per burst minus one, divided by the burst duration. Instantaneous firing frequencies were taken as the reciprocal of the interspike interval. For calculating phase, the beginning of the PD burst was used as a reference and all times were normalized to the cycle period.

### Preparation and Application of the Dyes

Two styryl dyes were used: RH795 (Pyridinium, 4-[4-[4-(diethylamino)phenyl]-1,3-butadienyl]-1-[2-hydroxy-3-[(2-hydroxyethyl)dimethylammonio]propyl]-, dibromide/172807-13-5; Biotium, Hayward, CA) and Di-4-ANEPPS (Pyridinium, 4-(2-(6-(dibutylamino)-2-naphthalenyl)ethenyl)-1-(3-sulfopropyl)-, hydroxide, inner salt/90134-00-2; Biotium, Hayward, CA). As Di-4-ANEPPS is highly lipophilic, the stock solution was prepared by diluting 5 mg of the dye in 1040 µl of 20% F-127 pluronic acid DMSO solution (Biotium, Hayward, CA; resulting dye concentration 10 mM). The stock solution was kept in darkness at room temperature.

For RH795, which is more hydrophilic, a 10 mM stock solution was prepared by diluting 5 mg dye in 854 µl of ultrapure water. The stock solution was kept in darkness at 4°C. For bath-application, both stock solutions were diluted in saline to the final concentrations of 0.05 mM, 0.1 mM and 0.2 mM. The range of concentrations was chosen according to previously published data [14], [16], [17], [33], [40]–[43].

We desheathed the STG to facilitate access of the dye to the STG neurons. A petroleum jelly well isolated the desheathed STG from other parts of the STNS (Fig. 1A). 50 µl of either Di-4-ANEPPS or RH795 were bath-applied to the well by using two pipettes - one to apply the dye on one side of the well and the other to remove the saline on the other side. The part of the STNS that was located on the outside of the well was constantly perfused with chilled saline (10–12°C) during dye application. Unless otherwise stated, dye application duration was limited to 30 minutes after which the preparation was rinsed with saline for about 10 minutes. During dye exposure the activity of the motor nerves was extracellularly recorded.

### Optical Imaging and Picture Processing

For recording fluorescence changes (‘optical imaging’) the MiCam02 imaging system and software (Brain- Vision Analyzer, BV-ANA; SciMedia Ltd, Tokyo, Japan) were used with the HR (High Resolution) camera (6.4×4.8 mm actual sensor size) set at either 384×256 pixel spatial resolution for high resolution photos or at 48×32 pixel spatial resolution. For the latter, a temporal resolution of 1.5 msec was chosen. Excitation light was provided by a CoolLED dual wavelengths system (535 nm and 470 nm; Yorktown Heights, NY). Used filter combinations were: excitation 540–560 nm, 560 nm beam splitter of 560 nm and 570–640 nm emission filter (Olympus, Hamburg, Germany) and 450–475 nm excitation, 500 nm beam splitter and 500–550 nm emission filter (Olympus, Hamburg, Germany). Fluorescence was detected with a 20×objective (WD 2.0 mm, NA 1.0, cc = water; Olympus Corporation, Tokyo, Japan) mounted on an upright fluorescence microscope (modified BX51, Scientifica, East Sussex, UK). Optical imaging was performed during illumination with excitation light of different intensities (5–20% of the maximum LED strength). For every LED light level two sessions of 24 seconds were recorded, each from a different area of the STG. The imaging was always started with the lowest light level (usually 5% of maximum LED strength) and then increased in 5% steps. For every concentration three different LED light strengths were used unless severe change in the motor output occurred. In the latter case, the LED light was not further increased. To compare the different recording conditions, the position of the microscope and cells was saved using the LinLab software (Scientifica, East Sussex, UK) that controlled the motorized microscope stage (SlicePlatform Pro 2000, Scientifica, East Sussex, UK).

Simple fluorescence level detection and merging of fluorescence pictures were performed using the LUCIA Measurement software (Laboratory Imaging spol. sec r.o, Praha, CZ) and a Vosskühler camera (Model CCD1300B, Allied Vision Technologies GmbH, Stadtroda, Germany) mounted on an Axiophot microscope (Carl Zeiss AG, Oberkochen, Germany). A standard 10×objective (Olympus, Hamburg, Germany) was used. Excitation light was provided by a 12 V halogen lamp 100 W (Carl Zeiss AG, Oberkochen, Germany), and either a 395–440 nm excitation filter with a 460 nm beam splitter and a 470 nm long pass emission filter or a 536–556 nm excitation filter with a 580 nm beam splitter and a 590 nm emission filter was used.

Measurements of mean grey values were performed using the ImageJ Launcher Software (version 1.45 s, Wayne Rasband, National Institute of health, USA; http://rsbweb.nih.gov/ij/). Final figures were created using Corel-Draw (version 12 for Windows, Corel Corporation, Ottawa, ON, Canada).

### Signal-to-noise Ratio

To determine the S/N, first the pyloric-timed fluorescence changes were recorded and averaged over multiple (>10) cycles of the pyloric rhythm. As trigger for the average, the first lateral pyloric neuron (LP) action potential of every LP burst on the extracellular recording was used and the time between two of those triggers was defined as the cycle period of the pyloric rhythm. Before averaging, the slow frequency components of the optical signal and baseline drifts due to thermal changes of the light source were removed using an internal function in Spike2 (DC-remove). In specific, the average value of time *t*−3 sec to *t* +3 sec was subtracted from the value at time *t*, resulting in the new output value. Minimum and maximum values of the signal were measured within one cycle of the average and the difference between those two values was defined as the amplitude of the signal. Most of the noise in the fluorescence signal possesses higher frequency components than neuronal events such as action potentials and synaptic inputs. Thus, for measuring the noise level we high-passed filtered the fluorescence signal to remove neuronal events (Spike2 DC-remove with a time base of p = 0.01 s). This eliminated most of the influence of slow membrane potential oscillations that typically occur in pyloric neurons. After the filtering the optical signal was averaged over multiple periods, triggered on the first LP spike on the extracellular recording and the standard deviation of the signal was then calculated for the duration of one period after the trigger. S/N was then calculated by dividing the previously measured amplitude by the standard deviation of the noise level.

### Statistics

For spreadsheet analysis, Excel (version 2010 for Windows, Microsoft) was used. Heatmaps were created using Gradient Contour Chart Add-In (gradient contour.xla). Further statistical tests were performed using GraphPad Prism (version 5.0 for windows, Graph Pad Software, Inc., La Jolla, CA, USA), OriginPro (version version 8, Origin lab Corporation, MA, USA), R-GUI Statistic (version 2.15.0, R foundation for statistical computing) or SigmaPlot (version 11 for Windows, Systat Software GmbH, Erkrath, Germany). Normally distributed data was plotted as mean ± SD. If data was not normally distributed, either median or all single values are given. “N” denotes the number of animals, while “n” is the number of trials. Linear correlations were tested with the Run test. In all figures significance is indicated using *(p<0.05), **(p<0.01), ***(p<0.001), ****(p<0.0001).

## Results

In order to investigate the staining abilities and properties of RH795 and Di-4-ANEPPS, both dyes were bath-applied separately to isolated STGs. During the first hour after dye application, pictures were taken every 10 minutes, then at 90, 120 and finally at 180 minutes. Possible pharmacological and illumination-associated toxic effects were assessed by monitoring the pyloric rhythm on the *dvn* (Figs. 1A, B).

### Staining Intensity

Both dyes stained the STG neurons and in general, fluorescence increased with application duration (Fig. 1C). Photos were taken with three different picture exposure times: Since Di-4-ANEPPS typically resulted in a stronger staining than RH795 (Fig. 1C), we chose longer exposure times for RH795 (100, 200 and 300 ms) than for Di-4-ANEPPS (50, 100 and 200 ms).

**Figure 2:**
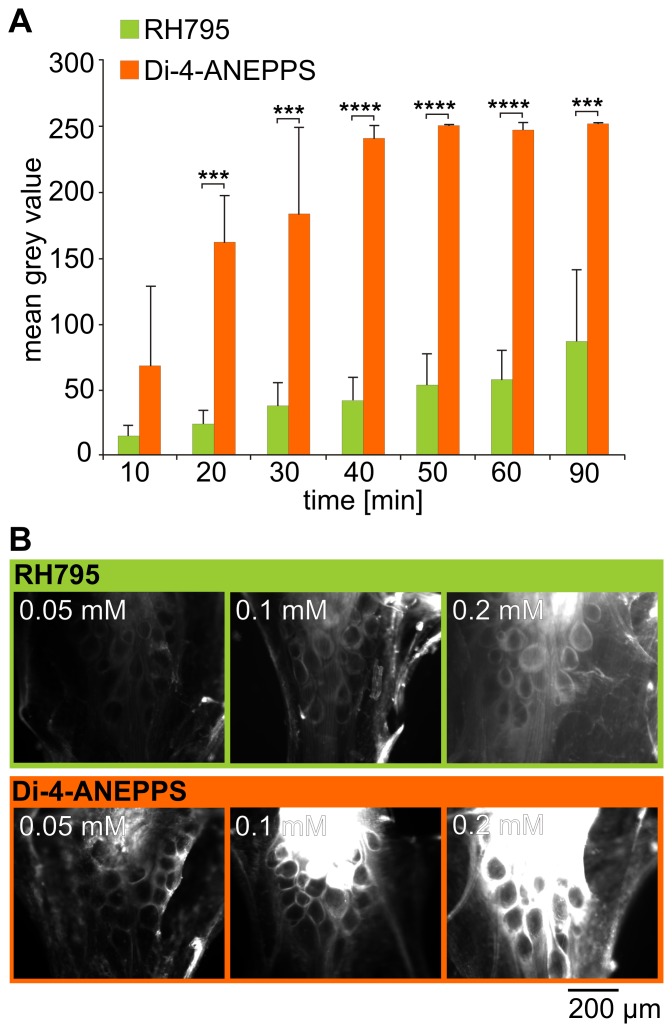
A. Di-4-ANEPPS exhibited higher fluorescence emission than RH795. Mean grey values of pictures taken from STGs after bath application of 0.05(green) or Di-4-ANEPPS (orange) (mean+SD, N = 3). Photos were taken with a camera exposure time of 200 msec. Dye type and time had significant influences on fluorescence emission (Two-way ANOVA, time: p<0.0001, dye type: p<0.01 for all measurement >10 minutes, Bonferroni multiple comparison test). B. Comparison of staining intensity for both dyes at different concentrations. Photos were taken 60 minutes after dye application.

The dyes were vastly different in their capacity to stain the STG. For example, the longest exposure time (200 ms) was necessary to obtain a reasonable fluorescence emission for RH795, even at the highest concentration used (0.2 mM), while the Di-4-ANEPPS staining intensity at this concentration exceeded the camera’s sensitivity range. In order to quantify the differences in staining intensity of the two dyes, we measured the brightness of the pictures as the mean grey value in the same quadrangular area of the neuropil in all preparations at an exposure time of 200 msec and a dye concentration of 0.05 mM. With the exception of 10 minutes after dye application, fluorescence intensity was always significantly higher for Di-4-ANEPPS than for RH795 (Fig. 2A; Two-way ANOVA, at least p<0.001 for each, N = 3). Duration of dye application also influenced staining intensity significantly (Two-way ANOVA, p<0.0001, N = 3), indicating that the staining intensity increased in a time-dependent manner for both dyes. This increase was faster for Di-4-ANEPPS than for RH795: After 40 minutes, the Di-4-ANEPPS staining reached 95.5±3.9% of its maximum value while RH795 staining reached only 47.1±21.1% of its maximum intensity (t-test; p<0.05; N = 3).

The initial analysis already indicated that the intensity of the staining may depend on the dye concentration. Figure 2B shows an example for each dye concentration used for RH795 and Di-4-ANEPPS taken after 60 minutes of dye exposure. On average, for Di-4-ANEPPS the staining intensity of 0.05 mM (27.20±3.71) was significantly different from 0.1 mM (173.68±63.5, N = 3; p<0.05) but 0.05 mM and 0.2 mM (126.37±98.11) and those at 0.1 mM and 0.2 mM were not. After 180 minutes, there was no more difference (p>0.05 for all). For RH795, there was neither a difference between the three concentrations at 60 minutes, nor at 180 minutes (p>0.05 for all; N = 3; 200 msec camera exposure time for RH795; 50 msec for Di-4-ANEPPS).

### Signal-to-Noise Ratio

**Figure 3:**
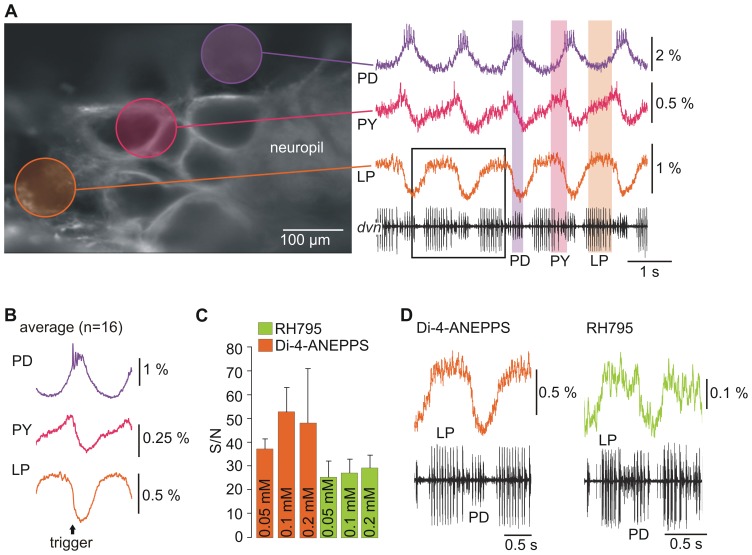
Optical recordings exhibited sufficient S/N to identify neuronal activity patterns. A. Original single-sweep optical recording of three different neurons (LP, PY, PD) after Di-4-ANEPPS application plus extracellular recording of the pyloric rhythm on the *dvn* (bottom trace). Analyzed areas are marked with circles in the high resolution photo (left). Areas included the cell membranes of the neurons analyzed. Scale bars mark the changes in fluorescence intensity. B. Cycle-triggered average (n = 16 cycles) of neurons shown in A. Averaging multiple cycles of optical imaging data improved S/N. C. The S/N was always higher for Di-4-ANEPPS, but independent of dye concentration. S/N was calculated using averaged data. D. Comparison of single-sweep LP optical recording in Di-4-ANEPPS (taken from inset in A.) and RH795 (different preparation).

Previous results demonstrated that the dyes differ in their ability to stain neurons. However, it was unclear whether this was reflected in the S/N during optical imaging. In general, the signal quality of optical signals from VSDs is lower than that of signals from intracellular recordings. In particular fast events such as action potetials can be difficult to detect (Fig. 3A; [16]). Thus, we tested which of the two VSDs produced the best S/N and which factors influence this ratio. S/N was measured for neurons that showed pyloric-timed fluorescence changes (averaged values; see Materials & Methods, Figs. 3A, B), using the same strength of illumination (15% of maximum LED strength) and the same concentration (0.1 mM) for both dyes. The concentration of 0.1 mM was chosen because it yielded the brightest staining for Di-4-ANEPPS, indicating that higher concentrations may not be suitable for measuring the S/N. Each dye generated a rather high S/N and optical signals were sufficiently large to separate and identify the pyloric neurons based on their slow wave oscillatory activity, even in single-sweep recordings (Figs. 3A, D). Averaging over multiple cycles further improved the S/N (by around a factor of 10; Figs. 3.A, B; see also [38]). On average, the S/N was higher for Di-4-ANEPPS (52.80±10.22; N = 4) than for RH795 (27.26±5.79; t-test; p<0.01; N = 4; see also Fig. 3C). This result also held true for the other dye concentrations tested, and similar to the results for staining intensity we did not find significant changes in the S/N when dye concentration was changed (Fig. 3C, One-way ANOVA: p>0.05 N = 4).

**Figure 4:**
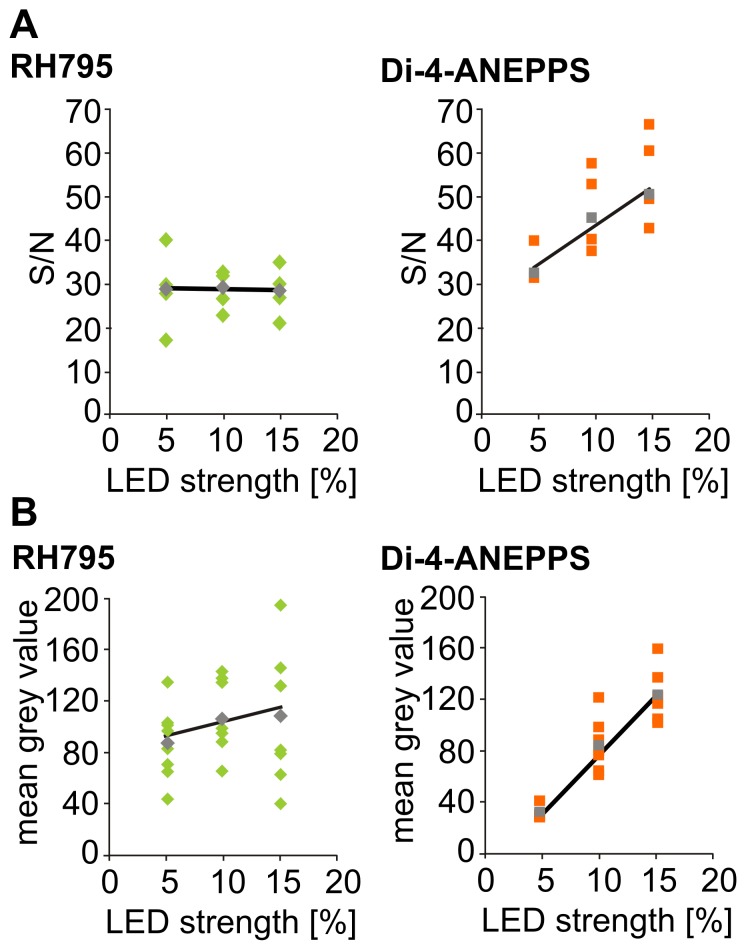
The S/N of RH795 is independent of LED strength. A. Left: S/N for RH795 did not change with excitation light strength (n = 12 trials in N = 3 animals, p>0.05). Right: S/N for Di-4-ANEPPS depended on excitation light strength (n = 10 trials in N = 3 animals, p<0.05). B. Fluorescence emission (mean grey values) increased with increasing excitation light strength for Di-4-ANEPPS (left; p<0.05; n = 22 trials from N = 4 animals), but not for RH795 (right; p>0.05; n = 23 trials from N = 4 animals).

The strength of the excitation light not only influences the fluorescence of VSDs, but has also been reported to affect the S/N [16]. Thus, we compared the S/N at different excitation light strengths (Fig. 4A). While for RH795 no significant change of the S/N with increasing excitation light strength was apparent (Run test: deviation of fit from linear was not significant; p = 1, R^2^ = 0.240; n = 12 trials from N = 4 animals; slope was not significantly different from zero, p>0.05), there was a significant correlation between S/N and excitation light strength for Di-4-ANEPPS (Run test: deviation of fit from linear was not significant, p = 1, R^2^ = 0.949; n = 10 trials from N = 4 experiments; slope was significantly different from zero p<0.05). One hypothesis why higher excitation light levels result in higher S/N for Di-4-ANEPPS is that increasing LED strength resulted in higher fluorescence emission for Di-4-ANEPPS stained preparations. Indeed, by plotting mean grey values, reflecting the fluorescence emission intensity, over LED strength (Fig. 4B) we found that higher excitation light strengths resulted in stronger fluorescence emission for Di-4-ANEPPS (Run test: deviation from linear not significant p = 1, R^2^ = 0.997; slope significantly different from zero p<0.05; n = 22 trials from N = 4 animals) but not for RH795 (Run test: deviation from linear not significant p = 1, R^2^ = 0.756; slope not significantly different from zero p>0.05; n = 23 trials from N = 4 animals).

In summary, the strength of the illumination affected the S/N for Di-4-ANEPPS, which may be explained by the concurrent increase in fluorescence emission. In contrast, the S/N was rather constant for RH795 and neither affected by dye concentration, nor by excitation light intensity, which could be due to the fact that none of these parameters caused an increase in fluroescence for RH795.

### Bleaching during Illumination

Bleaching of fluorescent dyes is a very common effect observed in imaging [18] and indicated by a reduction of staining intensity over time. Since the S/N can depend on the fluorescence emission, it is likely that bleaching has negative effects on the signal quality. Consequently, the stability of the dye should be considered as a criterion for choosing which dye to use, in particular for long-term recordings. We tested whether RH795 and Di-4-ANEPPS show differences regarding the level and speed of bleaching. Ganglia were stained for 24 hours with either Di-4-ANEPPS or RH795 to achieve a strong staining of neurons, and then rinsed with saline to avoid further influences of the dyes. Preparations were then illuminated for 60 minutes with constant light strength. Pictures were taken every 10 minutes to document the bleaching progress.

**Figure 5:**
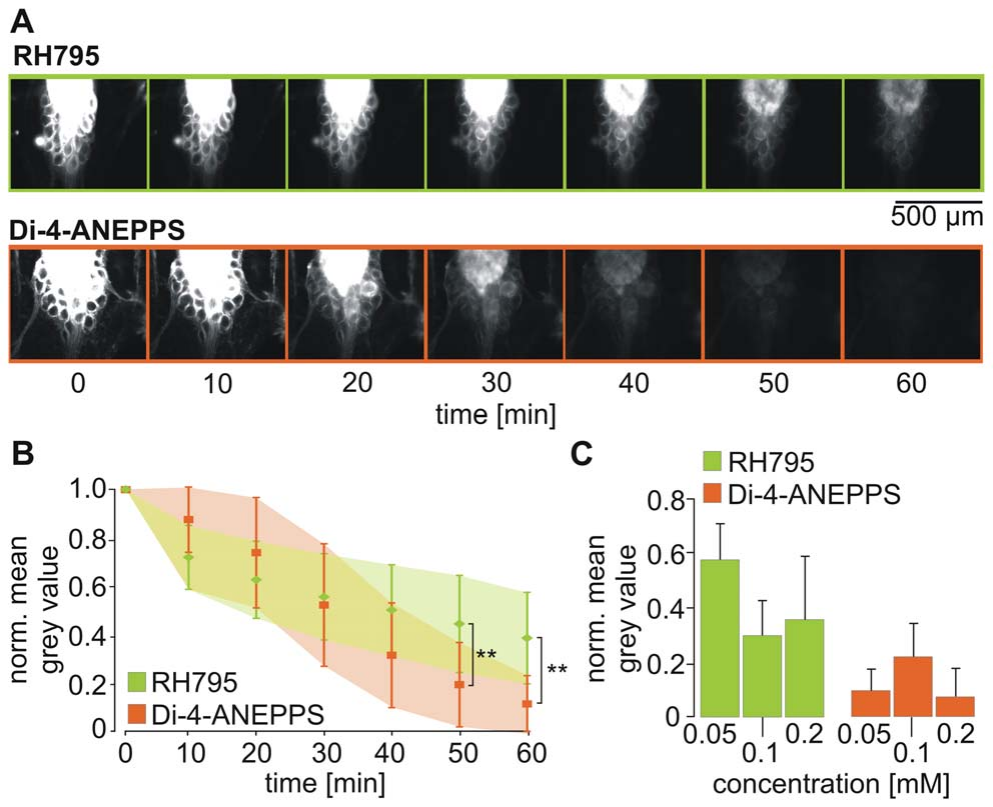
Di-4-ANEPPS and RH795 showed bleaching over time. A. Photos of STGs taken every 10 minutes during 60 minutes of constant illumination. Ganglia had been stained for 24 hours with 0.1(top) or 0.05 mM Di-4-ANEPPS (bottom) prior to light exposure. Camera shutter speed was 200 msec for RH795 and 100 msec for Di-4-ANEPPS. B. Bleaching was time-dependent for both dyes (Two-way ANOVA: p<0.0001; N = 12). Di-4-ANEPPS bleached faster than RH795 (Two-way ANOVA: p<0.05; N = 12). Mean grey values were normalized to the maximum and plotted over the duration of illumination. C. Comparison of the normalized mean grey values after 60 min of illumination, for all concentrations used (green: RH795; orange: Di-4-ANEPPS).

Picture exposure times were adjusted at the beginning of each experiment (values between 100 msec and 300 msec), depending on the strength of staining, and then kept constant for all pictures taken during these 60 minutes. Both dyes showed bleaching in a time dependent manner (Fig. 5A). After 60 minutes of constant illumination, STG somata were no longer visible in Di-4-ANEPPS stained ganglia. As a measure for decreasing fluorescence mean grey values of all somata in the STG were calculated and normalized to the brightest picture in each experiment (usually the first picture). Figure 5B shows that both dyes bleached significantly over time (Two-way ANOVA: p<0.0001; N = 12; all concentrations were pooled) and that the process of bleaching was significantly faster for Di-4-ANEPPS (Two-way ANOVA: p<0.05; N = 12). After 50 minutes and 60 minutes, normalized mean grey values of RH795 stainings were significantly higher than those of Di-4-ANEPPS stainings (Bonferroni multiple comparison test: p<0.01; N = 12).

We also tested if different dye concentrations were affected differently by bleaching (Fig. 5C). Interestingly, and in contrast to our expectation that higher concentrations would bleach faster, there was no significant effect of concentration on the speed of bleaching (Fig. 5C; One-way ANOVA, N≥3 for all concentrations, p>0.1). Yet, Di-4-ANEPPS bleached faster than RH795 and since it had been reported that bleaching affects the S/N [17], experiments with Di-4-ANEPPS are more limited by time than those carried out with RH795.

### Internalization of Dyes

**Figure 6:**
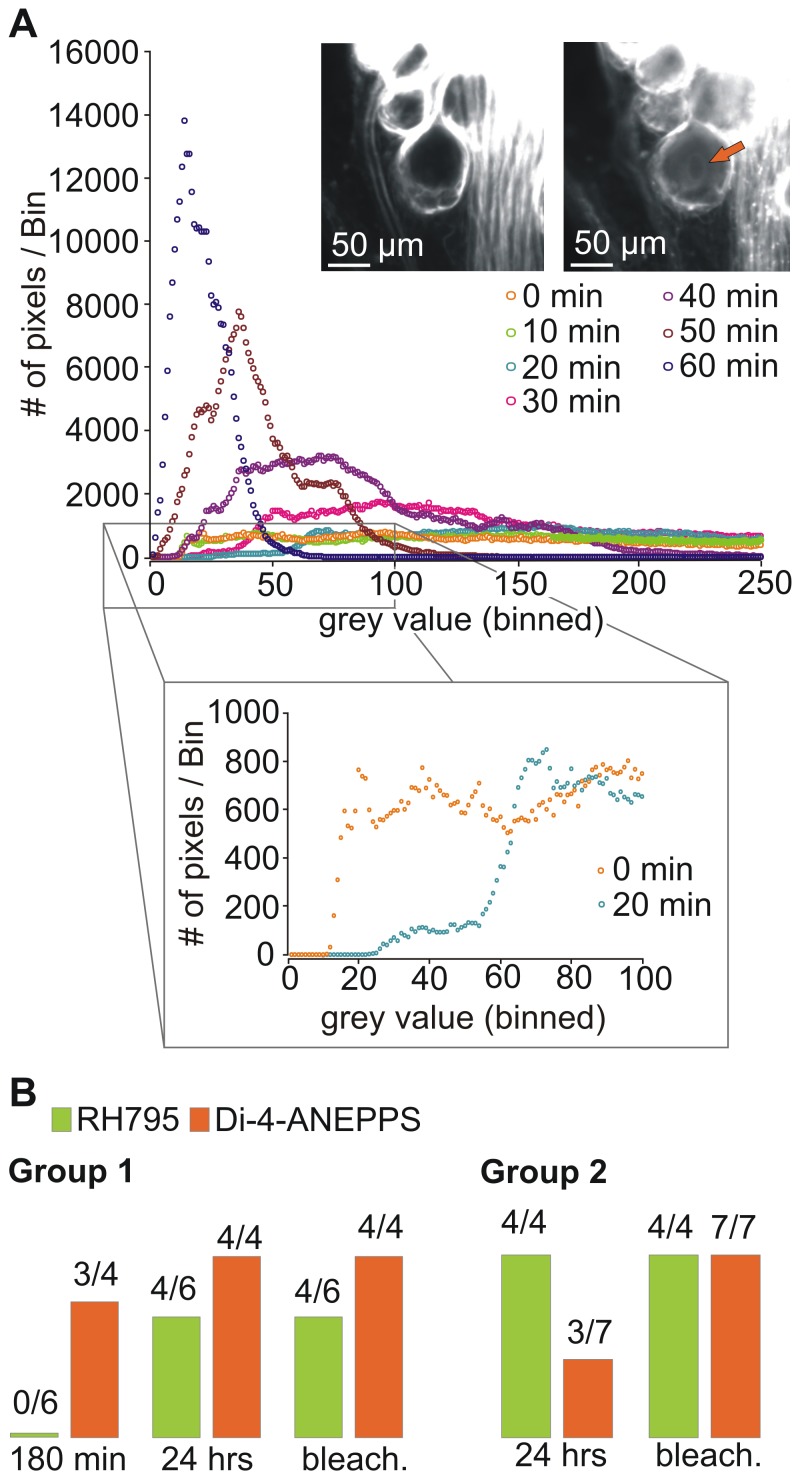
Both dyes were internalized during constant illumination. A. Di-4-ANEPPS was bath-applied for 24 hours and then washed out. Photos were taken every 10 minutes for 1 hour. The plot shows the frequency density of pixel brightness of the somata area. While in general the frequency of smaller grey values increased over time (due to bleaching), there were fewer dark pixels in the 20 minutes picture (blue) than in the initial picture (orange; bottom inset) as indicated by the shift of the frequency density to the right. No fluorescence: grey value of 0; highest fluorescence: grey value of 255. Binwidth: 1. For display purposes only bins 1 to 250 are shown. Top inset: Photos showing neuron staining immediately after dye wash-out and after 20 minutes of illumination. Immediately after washout cell bodies are dark while their surrounding is brightly stained. After 20 minutes of illumination the cell cytoplasm shows fluorescence and the cell nucleus is visible (orange arrow). B. Photos were analyzed and internalization was noted if at least the nucleus of one cell was visible. Group 1: Preparations that had been used for 180 minutes staining experiments (including illumination) (RH795: N = 6, Di-4-ANEPPS: N = 4). Photos were inspected for signs of internalization after the 180 minutes of staining experiments, after 24 hours staining and after bleaching. Group 2: Preparations were stained for 24 hours. Internalization was measured after the staining process and then again after 1 hour of bleaching (RH795: N = 4, Di-4-ANEPPS: N = 7). Group 1 Di-4-ANEPPS values after bleaching and group 2 RH795 values after bleaching were included for the sake of completeness (all preparations had shown internalization in the previous experimental step already).

An additional factor that limits experimental time - besides bleaching of the dyes - is the fact that cells can internalize VSDs into their cytoplasm [44]. Indeed we found indications that this also happened in our experiments. RH795 and Di-4-ANEPPS initially only bind to neuronal membranes and stain the cell borders (and axons). Figure 6A (inset) shows a photo that was taken after 24 hours of staining with Di-4-ANEPPS, but before light exposure. The specific binding to the cell membrane is characterized by a bright staining of the cell border, while the middle part of the cell appears dark. After 20 minutes of constant illumination, however, the inner part of the cell appears brighter and the nucleus is visible (arrow in Fig. 6A, inset) indicating that the cell internalized the dye into the cytoplasm [17], [44]. To quantify dye internalization we used luminosity histograms [17]. In these histograms, pixels of a picture are distributed into different brightness classes and their frequency densities are shown. Figure 6A shows an example for Di-4-ANEPPS. Each color represents the frequency density of pixel brightness of one picture taken at a particular time point during the bleaching process. In general, the area of highest density shifts to the left when plots are compared over time. This shift indicates that the number of dark pixels of the picture was increasing, reflecting the bleaching process of the dye. However, when the plot made from the initial picture (0 min, orange) is compared to the plot made from the picture taken 20 minutes later (blue), a shift to higher intensity can be observed. The shift from left to right is caused by a spread of the fluorescence, away from the cell border and into the cell body. Since the dye had been washed out before illumination had been started, the observed shift could not have been caused by additional dye molecules binding to the membrane. Rather, it indicates the internalization of the dye.

For analysis, we individually checked each photo for signs of internalization, namely the spread of the dye into the cell body and the appearance of the nucleus, and determined the point in time at which internalization first occurred. In four preparations, we confirmed the individually determined time of internalization by the shift of the frequency density plot to brighter pixels (N = 4). In total, N = 10 preparations were tested for RH795 and N = 11 for Di-4-ANEPPS. Preparations were divided into two groups: (1) Preparations that were originally used for testing the success of the dye staining and (2) preparations that were exclusively stained for bleaching experiments and had not been illuminated during the 24 hours period of staining. All preparations were checked for internalization at different time points. Group 1 was first analyzed after 180 minutes of dye application, again after 24 hours and after another hour of bleaching. Group 2 STGs were checked after the 24 hour of staining and then again after one hour of bleaching. The proportion of ganglia of both groups that had internalized the dye into the cytoplasm at those three time points is shown in Figure 6B. For RH795, no ganglion showed internalization during the initial 180 minutes of staining (N = 6). However, after 24 hours, 4 of the 6 preparations showed internalization (67%). No additional ganglion internalized RH795 during the subsequent bleaching process. In contrast, all preparations that had been stained for 24 hours (but had not been exposed to light; N = 4) had internalized the dye.

Most Di-4-ANEPPS stained ganglia showed internalization already after initial staining (and illumination) procedure (75%, N = 4). After 24 hours, all of the ganglia from these experiments had internalized the dye, compared to three out of seven from those preparations which had only been stained for 24 hours, but not been perturbed otherwise (43%, N = 7). After the bleaching process *all* Di-4-ANEPPS stained ganglia had internalized the dye.

In summary, internalization for RH795 took place during the 24 hour overnight staining period without illumination. Therefore, this process seems to depend more on exposure to the dye than on illumination. This is also supported by the fact that no ganglion was observed to internalize dye during the bleaching process. In contrast to Di-4-ANEPPS, not all ganglia stained with RH795 showed internalization at the end of the experiments, indicating that Di-4-ANEPPS is internalized faster than RH795. This can also be seen in the higher proportion of ganglia that internalized Di-4-ANEPPS during the initial 180 minutes staining and illumination. Most of the Di-4-ANEPPS uptake took place during a time when the ganglion was illuminated (staining and bleaching experiment) which indicates that excitation with light promotes the internalization process for Di-4-ANEPPS.

### Pharmacological Effects and Photodynamic Damage Caused by RH795 and Di-4-ANEPPS

**Figure 7:**
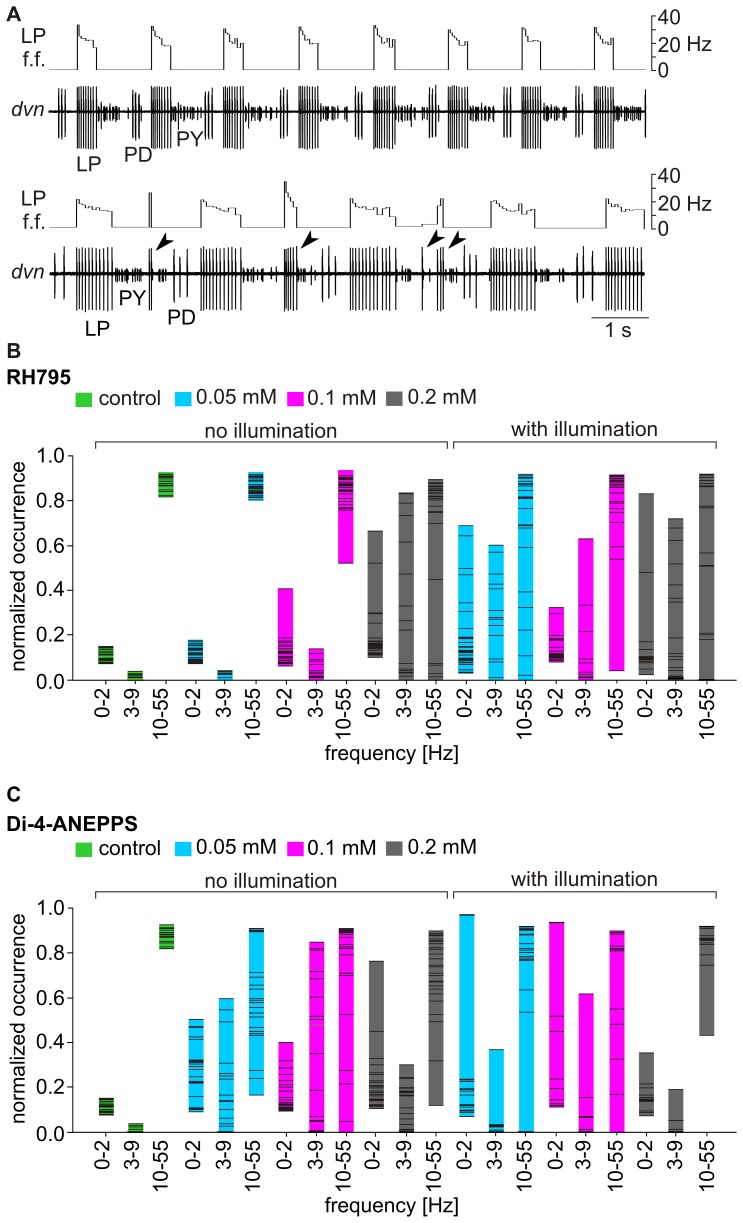
Dye toxicity and phototoxicity increased over time. A. Example of an extracellular recording of the triphasic pyloric rhythm on the *dvn* showing the activity patterns of PD, LP and PY and plot of instantaneous firing frequency (f.f.) of LP. Top: saline control. Bottom: after 180 minutes exposed to Di-4-ANEPPS (0.1 mM). Arrows indicate extra LP spikes. B. The distribution of LP instantaneous spike frequencies changed over time. The frequencies ranges given are 0–2 Hz, 3–9 Hz and 10–55 Hz. The firing frequency distribution changed for RH795, between different conditions (saline control and 0.05 mM, 0.1 mM and 0.2 mM RH795) and for illuminated and non-illuminated preparations (N = 3, measurements every ten minutes for one hour, then at 90 minutes, 120 minutes and 180 min). Each bar includes data from three experiments. C. Similar to B., for Di-4-ANEPPS.

Binding of dyes to neuronal membranes can have pharmacological effects on neuronal activities [45], [46] and thus alter the functioning of neurons. Furthermore, in the presence of illumination, reactive single oxygen and other radicals can occur [19], [23], [47] and harm the cells. These effects limit experimental time. Since it had been reported that toxicity increases with dye concentration in various systems and for various dyes [48], we tested whether RH795 and Di-4-ANEPPS show toxicity in the STNS for all concentrations used. To characterize possible toxic effects on the motor output of the system, changes in the extracellularly recorded pyloric rhythm were assessed during all experiments. In healthy conditions, the pyloric rhythm shows stereotyped and stable activity patterns. In particular, phase relationships [49]–[51], and firing frequencies [49] as well as spike phasing [52] remain constant over extended periods of time. This stability of the rhythm provides the chance to study toxic effects not only for single neurons, but also on a network level. The pyloric rhythm is triphasic and can be monitored by the activities of three types of neurons that appear in different sizes on the extracellular recording of the *dvn*: LP, which is the biggest unit on the *dvn*, pyloric constrictor neurons (PYs), which are the smallest units and the PDs [24]. Since the VSDs caused a broad range of changes in motor activity, we used three measures to analyze changes in the rhythm: (1) Changes in the cycle period, i.e. whether the rhythm sped up or slowed down, (2) the occurrence of additional spikes or the lack of spikes in a burst and (3) the intraburst firing frequencies. Since it was difficult to identify different neurons on the *dvn* when preparations became arrhythmic (and hence some of the neurons showed tonic instead of rhythmic activities), we used the LP action potentials, which were usually easy to identify by their large action potential amplitudes. We assessed the named changes in the rhythm by measuring the instantaneous firing frequencies of LP. In control conditions, two frequency ranges occurred: (1) The LP intraburst frequency that is usually found between 10 Hz and 55 Hz [49] and (2) a slower frequency between 0.5 Hz and 2 Hz. The latter is produced by repetition of bursts and represented the gap between two subsequent LP bursts [24] and thus roughly the cycle period of the rhythm. If action potentials in a burst are missing or additional action potentials are elicited in-between bursts, additional frequencies occur in-between those two frequency bands, i.e. between 3 Hz and 9 Hz. Figure 7A shows the pyloric rhythm in control conditions, at the beginning of an experiment (immediately after dye application). Below, the pyloric rhythm is shown after 180 minutes of Di-4-ANEPPS application (without illumination). The rhythm had slowed down, the total number of spikes was reduced and additional LP spikes occurred in-between regular LP bursts. As the preparation was not illuminated and temperature was kept constant, these changes in the rhythm must have been caused by the pharmacological effects of Di-4-ANEPPS. Figure 7B shows the distribution of LP frequencies over 180 minutes for different treatments (different dye concentrations, with or without illumination, respectively). To compare these treatments, the relative occurrence of the three LP frequencies bands (0–2 Hz, 3–9 Hz and 10–55 Hz) was calculated every ten minutes for the first hour, then at 90 minutes, 120 minutes and 180 minutes. The occurrence of frequencies was then plotted for all measurements. Different treatments were labeled in different colors. In general, short bars indicate that distribution was stable over 180 minutes while larger bars show big changes in the distribution of frequencies. The figure summarizes data from three animals for each treatment. Control conditions are illustrated by the three bars on the left (green). Here, only saline was applied to the STG. As expected for the control condition, bars are small indicating that frequency distribution remained rather unchanged over 180 minutes. Small bars also indicate that in control conditions different animals showed similar frequency distributions and that most frequencies occurred between 0–2 Hz or between 10–55 Hz. Besides chemical toxicity of VSDs (which affects neurons by their mere presence), phototoxicity during illumination may occur. In order to test this, preparations treated with dye were divided into two groups. One group was kept in the dark so that changes in the rhythm were caused only by the chemical effects of the dyes (Fig. 7B, left nine bars), while the other group was illuminated by taking pictures, as it would be the case during optical imaging (Fig. 7B, right nine bars). For both, RH795 and Di-4-ANEPPS, bars increased from left to right in non-illuminated and illuminated preparations, i.e. with increasing dye concentration. It can be concluded that changes in rhythmical activity are concentration-dependent for both dyes. Furthermore, frequencies in the 3–9 Hz class occurred, which were rarely present in control conditions. When preparations were illuminated, the number of frequencies that belong to this class increased compared to the control situation for all concentrations of RH795. For non-illuminated preparations only 0.2 mM RH795 caused an increase in that class. For Di-4-ANEPPS, all concentrations for both illuminated and non-illuminated preparations showed an increased number of additional frequencies over 180 minutes of the experiment duration compared to the control situation. These additional frequencies can be explained by two phenomena that were observed during experiments: (1) Missing of single spikes in the LP burst. This left a gap that resulted in longer interspike intervals between LP spikes and thus caused occurrence of lower instantaneous frequencies. (2) Switching of LP from rhythmic to tonic activity, which also results in lower LP firing frequencies.

**Figure 8:**
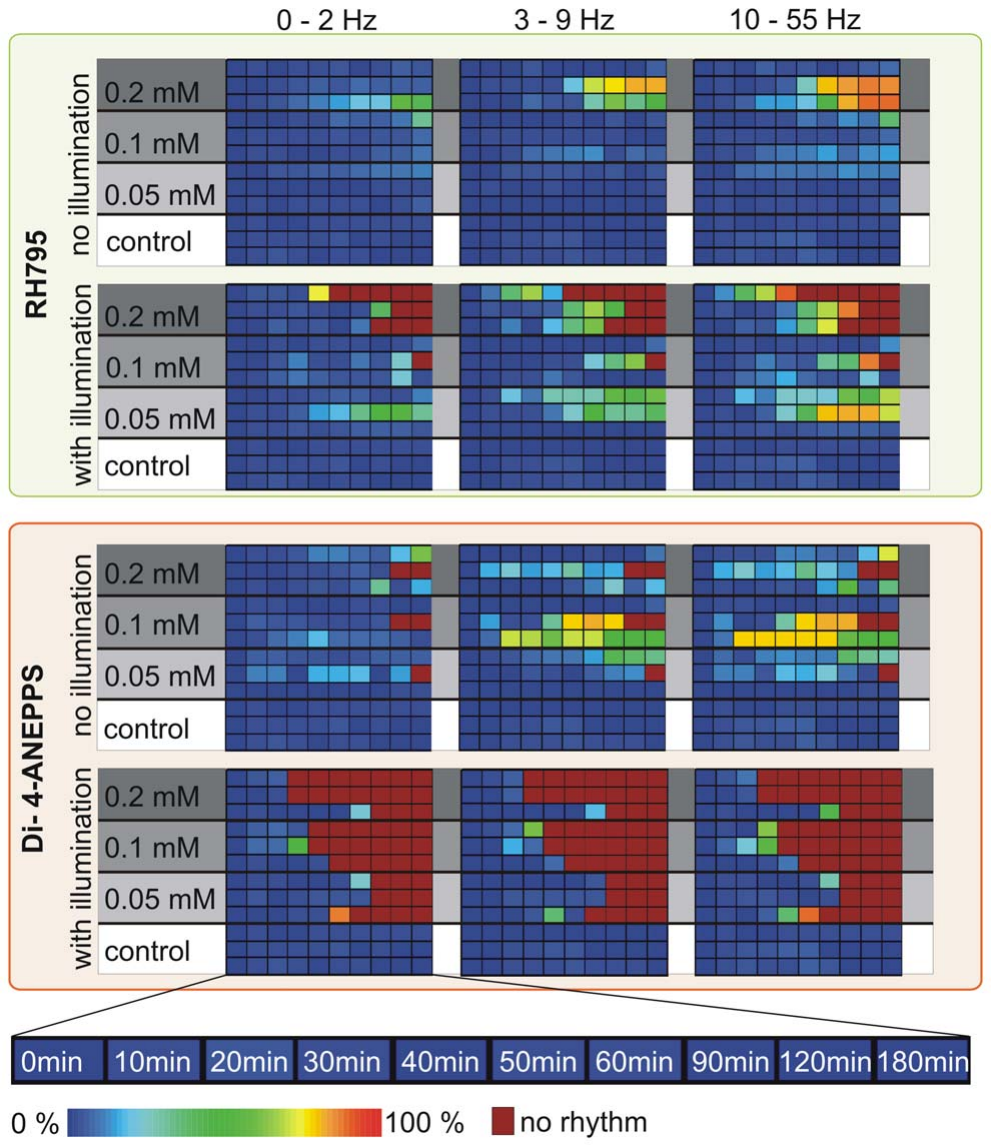
RH795 had weaker pharmacological and phototoxic effects than Di-4-ANEPPS. Separate ganglia were used for each dye and concentration. During the experiment the pyloric rhythm was recorded extracellularly from the *dvn* and the instantaneous firing frequencies of LP were monitored. Frequencies were measured for 60 seconds each, every 10 minutes for one hour, than at 90 minutes, 120 minutes and 180 minutes. Colors code differences from a control measurement before dye application. Cool colors indicate small deviations from control and hot colors represent large deviations. Data from one animal is represented in one line, from left to right. Preparations that experienced the same treatment are highlighted with the same grey boxes (N = 3). The saline control is always represented by the bottom three lines highlighted in white. Firing frequency classes are shown by columns (left to right: 0–2 Hz, 3–9 Hz and 10–55 Hz).

Differences between illuminated and non-illuminated preparations were clearly visible for RH795 stainings (Fig. 7B). Bars belonging to recordings from illuminated preparations are in general larger than those from non-illuminated ganglia, indicating that toxic effects were stronger when preparations were exposed to light. For Di-4-ANEPPS, differences between those two groups were smaller. Figure 8 illustrates this in more detail: Here, the relative changes from control conditions (before dye application) are shown. Colors code the level of change compared to controls. Cold colors, such as blue, indicate no or small changes while warm colors, such as red, indicate strong changes. If spikes were not clearly identifiable or preparations showed only sporadic spike activity, dark red color was used to indicate extreme changes that could not be quantified. Data from one animal is represented in one line from the very left to the very right box, and preparations that experienced identical treatment are highlighted with the same grey boxes (N = 3). Control conditions are always represented by the bottom three lines, highlighted in white. Experiments are subdivided in illuminated (bottom three boxes) and non-illuminated preparations (top three boxes). Firing frequency classes are shown by columns. The left column of boxes shows the frequency class 0–2 Hz representing interburst frequencies, the middle column (3–9 Hz) and right column (10–55 Hz) include all intraburst frequencies.

For the control situation in which animals were only treated with saline, all data points shown are blue illustrating that the firing frequency distribution did not change within 180 minutes of the experiment, when compared to the beginning of the experiment. For RH795, without illumination, only preparations that were treated with 0.2 mM concentration were affected (top three rows). For illuminated preparations all concentrations caused changes over time (bottom three boxes, from left to right in each box). The effects were stronger for higher concentrations (compare bottom rows to top rows) in both categories. Comparing non-illuminated with illuminated preparations, it appears that illumination caused severe changes of the rhythm. For Di-4-ANEPPS, similar results were obtained: Both, a mere concentration-dependent effect, as well as a worsening due to illumination could be detected (bottom six boxes). Comparing Di-4-ANEPPS with RH795 shows that Di-4-ANEPPS had stronger effects on the rhythm than RH795. Thus, toxicity depended on concentration, is generally higher for Di-4-ANEPPS than for RH795 and increased when preparations were illuminated.

**Figure 9:**
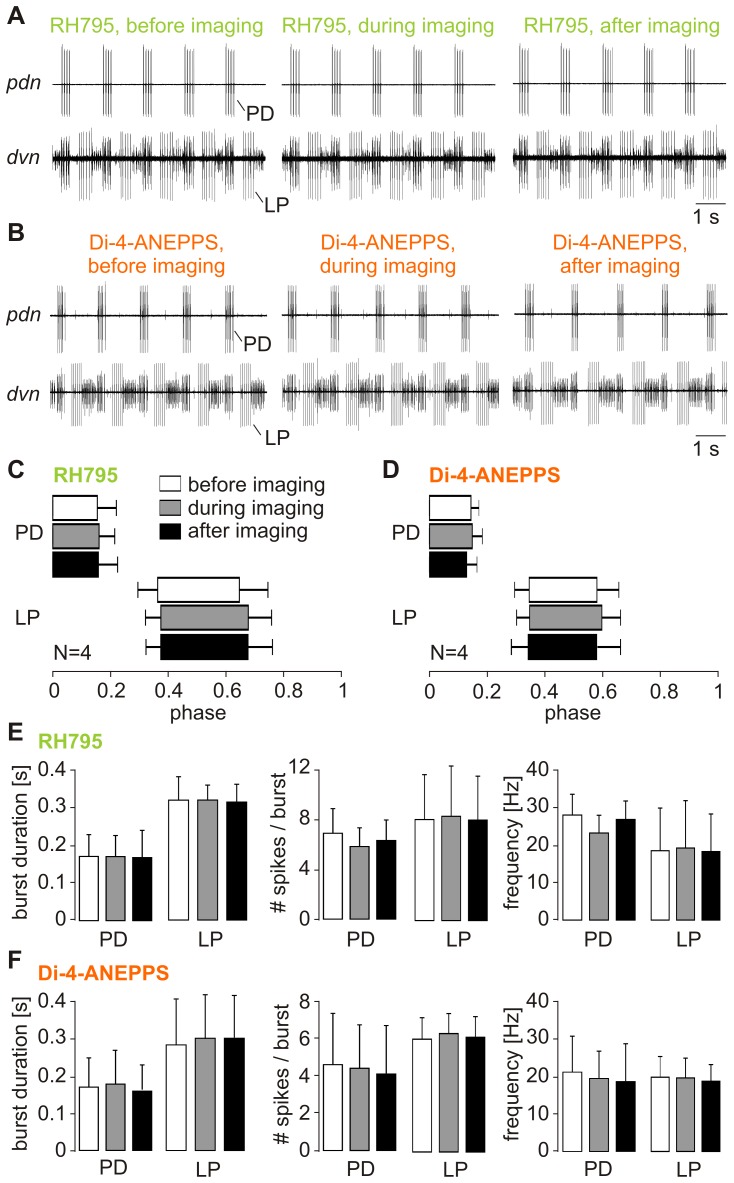
The pyloric rhythm was unaffected during imaging. A. There were no short-term influences on the pyloric rhythm during illumination in RH795 stained preparations. Original recordings of the pyloric dilator nerve (*pdn*) and the *dvn* before, during and after illumination. Spike activities and phasing of the pyloric neurons were virtually identical in all conditions. B. Original recordings of *pdn* and *dvn* in a preparation that had been stained with Di-4-ANEPPS. There was no apparent change in the pyloric rhythm during illumination. C. Phase diagram showing the relative contribution of PD and LP neurons to the pyloric cycle before, during and after illumination in RH795. There were no significant differences (One-way ANOVA, p>0.6 for all comparisons, N = 4). D. Phase diagram for illumination in Di-4-ANEPPS. Illumination did not affect the phasing of LP and PD (One-way ANOVA, p>0.6 for all comparisons, N = 4). E. Comparison of burst duration (left), number of spikes per burst (middle) and intraburst spike frequencies (right) of PD and LP before, during and after illumination in RH795 stained preparations. No significant differences were found (One-way ANOVA, p>0.9 for all comparisons, N = 4). F. Similar comparison in preparations that were stained with Di-4-ANEPPS (One-way ANOVA, p>0.9 for all comparisons, N = 4).

It had been reported that illumination of Di-4-ANEPPS-stained STG neurons can temporarily alter the pyloric motor pattern [33], and in particular the speed of spiking and phasing of the neurons. The lowest concentration (0.05 mM) caused the weakest toxicity in our experiments. To test whether transient effects during illumination were also obvious at this concentration and at illumination strengths that resulted in a sufficient S/N, we compared the pyloric rhythm before, during and after illumination for both dyes (Figs. 9A,B). There were no effects on the rhythm. Cycle period was neither affected by illumination in RH795 (pre-control: 1.46±0.40 s, illumination: 1.36±0.31 s, post-control: 134±0.29 s, One-way ANOVA, p>0.5, N = 4) nor in Di-4-ANEPPS stained neurons (pre-control: 1.16±0.38 s, illumination: 1.17±0.38 s, post-control: 122±0.37 s, One-way ANOVA, p>0.6, N = 4). Also, phasing of the neurons remained stable during illumination (Figs. 9C,D; One-way ANOVA, p>0.6 for all comparisons, N = 4), as did burst durations, the number of spikes per burst and firing frequencies of PD and LP (Figs. 9E,F; One-way ANOVA, p>0.9 for all comparisons, N = 4).

## Discussion

Voltage-sensitive dyes are used to detect membrane potential changes in many different systems and various cell types [53]. When recording from neurons, optical imaging can complement electrophysiology in cases where the latter is insufficient, but detailed neuronal resolution is required. Yet, optical imaging comes with disadvantages. We focused on the use of VSDs for the STNS, a well-characterized model system for central pattern generation [24], [25] for two reasons: First, its stable rhythmic motor patterns allows direct assessment of functional output of a whole motor circuit (instead of just single cells) and thus creates a system for characterizing possible pharmacological or light-induced effects on the neurons. Second, recent research in pattern generation has moved towards investigating homeostatic processes that act over many hours and sometimes even days [31], [32], [54], [55]. Recording membrane potential changes with sharp intracellular electrodes over such extended periods of time is challenging and often harmful for neurons, since it bears the risk of damaging cell membranes. It is also a time consuming procedure and the number of cells that can be recorded simultaneously is limited (4–6 neurons, with the notable exception of [56]). VSD imaging offers the opportunity to simultaneously study multiple neurons and their interactions in different behavioral conditions [16]. In principle, the activity of as many neurons as are visible in the field of view of the camera can be recorded. However, signal quality will be determined by the optics of the microscope and the sensitivity of the camera and is lower compared to intracellular recordings with glass microelectrodes.

Fast VSDs, like RH795 and Di-4-ANEPPS, report membrane potentials in a high spatiotemporal resolution, often with time constants of less than 1 µs [57]. For the dyes discussed here, this is due to the fact that potential changes result in a quick charge shift inside the chromophore that causes differences in their fluorescence characteristics [58] which are directly correlated to the membrane potential. VSDs are thus superior to Calcium-sensitive dyes that report the presence of free calcium in the cytosol, which limits their temporal and spatial resolution [59], [60]. Calcium-sensitive dyes also act as calcium buffers and chelate calcium [61] and could therefore interfere with calcium homeostasis inside the cells.

### Different Voltage-sensitive Dyes - different Characteristics

The successful use of the optical imaging technique mainly depends on the choice of a suitable VSD. The first concern is typically whether the dye is able to stain neurons in the system of choice. Di-4-ANEPPS and RH795 stained neurons in the STG at all concentration used. Staining intensity was higher and staining proceeded also faster for Di-4-ANEPPS compared to RH795. Both dyes belong to the group of styryl dyes but their molecular structures are different. Di-4-ANEPPS has longer alkyl chains that are attached to the chromophore than RH795 which makes Di-4-ANEPPS more lipophilic [17]. Since affinity and binding to the membrane depends on the length of these alkyl chains, this gives Di-4-ANEPPS a higher affinity to membranes [41]. Over the past decade different Chimeric dyes (Di-n-ANEPPDHQ) have been synthesized [17] that combine various properties of the dyes used in our study. Future experiments need to show whether these chimeric dyes are more suitable for long-term recordings and whether they show good staining intensity and S/N in STG neurons.

There were limits for staining intensity. Increasing Di-4-ANEPPS concentration above 0.1 mM did not further increase staining intensity. This could be due to saturation of the membrane with dye molecules. In fact, a further increase may lead to a weaker staining since molecules that are already present in the membrane could be displaced rapidly by other dye molecules. This constant exchange could in turn diminish the intensity of the fluorescence emission. Alternatively, at high concentrations, dye molecules may interact with themselves or with the solution, in which they are dissolved. In particular in salt water animals such as the crab species used here, dyes are diluted in a highly polar saline solution, and it is conceivable that amphiphile dye molecules form micelles, similar to other amphiphile molecules [62]. If lipophilic alkyl chains are locked in micelles, they are not available for insertion into the membrane and, consequently, the number of dye molecules bound to the membrane could be reduced.

### The Signal-to-noise Ratio is Influenced by Many Factors

In general, VSD-recordings show smaller S/Ns than intracellular recordings [9], [16], [63], [64] and much effort is put into the improvement of the signal quality [12], [18], [41], [53], [65]. The signal quality depends on the hardware properties of the particular recordings system, such as transmission efficiencies and band pass filter width and absorption characteristics of the detector. It can therefore vary between different recording systems. Common to all systems, however, is that the S/N depends on signal amplitude and the noise generated during the detection of the signal, as well as on the intrinsic capacity of the dye to sense membrane voltage. In our hands the S/N was well-suited to study neuronal activity in the STG. STG neurons could be identified based on their slow fluorescence waveform, a prerequisite for studying the central pattern generators in the STG. We found a significantly better signal quality for Di-4-ANEPPS compared to RH795 when tested at the same concentrations and excitation light strength. The S/N for RH795 was about half of that achieved for Di-4-ANEPPS. The S/N for RH795 was not affected by the excitation light level, while it was for Di-4-ANEPPS. Signal amplitude is highly sensitive to small changes in dye structure and depends on the hydrophobicity of the dye [47]. Thus, it is conceivable that the obtained differences between RH795 and Di-4-ANEPPS signals are determined by their molecular structure and were not the result of specific experimental arrangements results correspond to previous observations for mercocyanine-oxazolone dyes that higher concentrations and light levels do not necessarily lead to better signal quality [9] and that for most dyes an optimum range for these parameters exists [16], [47]. Increasing these parameters beyond this optimum could further facilitate pharmacological toxicity instead.

### Characteristics of Dyes that Limit the duration of Optical Imaging

Bleaching as well as pharmacological and photo-induced damage can diminish signal quality [47], [48], [66], and since they increase over time they are particularly problematic for long-term recordings. The reduction in signal quality during bleaching emerges because photolytic products of bleaching appear to be unexcitable [9]. While the exact mechanisms that cause bleaching are still unknown, it is suggested that the conjugated carbon chain of the dye molecules is broken down [17]. Since there are differences between the conjugated carbon chains of RH795 and Di-4-ANEPPS, it was likely that both dyes also show different bleaching characteristics. Indeed, Di-4-ANEPPS bleached faster than RH795. Bleaching can easily be mixed up with a wash-out of the dye, i.e. the departing of the dye molecules from the neuronal membrane to the aqueous phase [17]. Yet, wash-out is inversely proportional to the length of alkyl chains [41] and as Di-4-ANEPPS has longer alkyl chains than RH795, it should show a slower wash-out than RH795. Thus, it is likely that the obtained decrease in fluorescence was indeed due to bleaching and not due to wash-out.

Since neither S/N nor bleaching depended on the concentration for RH795, the lowest concentration (0.05 mM) appears suitable for the use in long-term experiments. Di-4-ANEPPS has the advantage of a higher S/N but also loses signal-quality quickly due to bleaching. Interrupting the experiment and adding fresh dye to the ganglion may solve this problem, since it had been reported that staining intensity can be recovered by repetitively applying fresh dye [9].

### Pharmacological and Photodynamic Damage

Bleaching and photodynamic damage are the most time-limiting factors for experiments with VSDs [67]. RH795 had previously been suggested to possess lower toxicity than ANEPP dyes [17], which made it particularly interesting for characterizing its long-term effects. Here, rather than using stimulus-evoked responses, we analyzed spontaneously active rhythmic motor patterns that were generated by a network of neurons. This allowed us to detect even subtle effects on the natural activity of the system. In agreement with these previous studies, we found that toxic and phototoxic effects of RH795 were smaller than those of Di-4-ANEPPS and that bleaching was slower as well. Long-term recordings over several hours showed that even after 180 minutes of simultaneous illumination of multiple neurons, the application of the lowest concentration of RH795 had only minor influence on the motor pattern output of the network. This means that not only on a single cell level but also on a network level, where toxic effects can accumulate, low concentrations of RH795 are suitable for long term recordings. Di-4-ANEPPS, on the other hand, had a clear effect on the rhythm at all concentrations, even without illumination. Our previous studies had indicated that a 12 hour exposure to Di-4-ANEPPS at a concentration of 0.05 mM does not affect the pyloric activity [33]. This apparent contradiction appears to be due to differences in the staining procedure: in this study, preparations were superfused with saline at physiological temperatures (10–12°C) during the dye application, while in our previous study overnight stainings at 4°C without perfusion were performed. As lower temperatures slow down all physiological processes, cooling of the preparation could have reduced the pharmacological effects. With illumination, severe influences on the motor pattern occurred as early as 20 minutes after dye application at the highest concentration.

Schaffer and colleagues [40] suggested that one of the mechanisms of toxicity is that dye molecules binding to membranes alter membrane properties such as fluidity, ionic conductances and capacitance. In hippocampal neurons, for example, even low concentrations of Di-4-ANEPPS modulate GABA receptors and potentiate their responses to low GABA concentrations [46]. GABA is also known to affect the gastric mill and pyloric motor patterns [68], [69]. GABA application can cause an inhibition of PD pacemaker neurons and therefore lead to disinhibition and tonic firing of the LP neuron. GABA is released by descending modulatory projection neurons (e.g. MCN1, [68]) but despite this, LP and PD neurons usually fire rhythmically. One of the phototoxic effects of Di-4-ANEPPS and RH795 was a change in LP firing frequency. Instantaneous firing frequencies between 3 and 9 Hz occurred, which indicated that LP stopped its typical rhythmic activity and was active tonically. This finding is in line with the idea that the sensitivity of pyloric neurons for GABA was increased.

It is noteworthy that changes in LP spiking activity served as a measurement for the healthiness of the preparation, but their origin remains unclear. In general, the activity changes must not necessarily originate from changes in intrinsic properties of the neuron itself, but could occur because of influences of the dye on other neurons, that are carried to LP *via* its network connections.

For both dyes, the lowest concentration allowed a period of imaging without major pharmacological or photodynamic damage (RH795 around 180 minutes, Di-4-ANEPPS around 60 minutes). Within this timeframe, illumination of preparations had no influence on the motor pattern (Fig. 9). Thus, RH795 and Di-4-ANEPPS allow optically recording STG neuron membrane potentials without influencing ongoing activities, and without immediate phototoxic damage. Previous reports using this system [33] suggested that illumination causes temporary excitation of the pyloric rhythm. We found that stronger excitation light induced quicker and more pronounced toxic effects on the motor pattern (Fig. 8) than lower excitation light strengths. It is thus plausible that stronger excitation light also causes temporary effects on the rhythm during illumination. While excitation light levels used here did not affect the pyloric rhythm, increasing excitation strength increased the S/N for Di-4-ANEPPS. In contrast, for RH795, no such correlation was found, indicating that for RH795 low concentrations and illumination strengths allow detecting optical signals with maximal signal quality without quick phototoxic damage and without impairing neuronal activities.

### Internalization

Besides bleaching and toxicity, the internalization of dye molecules into the cytoplasm limits experimental time. The mechanisms of internalization are not well studied, but when the dye is internalized dye molecules are removed from the excitable membranes [17]. This process is therefore accompanied by a decrease of the S/N. At the same time, phototoxicity increases as the dye molecules have access to the cell organelles [17]. For RH795, internalization mainly occurred during 24 hour overnight staining, which was a period without illumination. In general, internalization of RH795 seemed to depend more on the time of exposure to the dye than on the illumination of the preparation. This is also supported by the fact that no RH795-stained preparation showed internalization during the bleaching process. In contrast to previous studies that report immediate internalization of RH795 by neurons of the submucous plexus of guinea pig [17], no STG neuron internalized RH795 in the initial 180 minutes, and even after more than 24 hours not all preparations showed dye internalization. Di-4-ANEPPS was internalized faster than RH795 and almost exclusively during the period of illumination. Faster internalization of Di-4-ANEPPS may partially be explained by the composition of the dye solution: Due to its lipophilic character, Di-4-ANEPPS has to be diluted in Pluronic acid and DMSO, two solvents which make the dye more likely to pass through membranes.

In summary, RH795 and Di-4-ANEPPS can be used for optical imaging in the STNS. They allow simultaneous recording of multiple neurons with good signal quality. While signal quality was better for Di-4-ANEPPS, toxic and phototoxic effects as well as bleaching were also enhanced. On the other hand, RH795 can be used in long-term experiments while causing minor effects on the motor pattern.
